# Modular Serial Flow Through device for pulsed electric field treatment of the liquid samples

**DOI:** 10.1038/s41598-017-08620-8

**Published:** 2017-08-14

**Authors:** Maša Kandušer, Aleš Belič, Selma Čorović, Igor Škrjanc

**Affiliations:** University of Ljubljana, Faculty of Electrical Engineering, Tržaška 25, SI-1000 Ljubljana, Slovenia

## Abstract

In biotechnology, medicine, and food processing, simple and reliable methods for cell membrane permeabilization are required for drug/gene delivery into the cells or for the inactivation of undesired microorganisms. Pulsed electric field treatment is among the most promising methods enabling both aims. The drawback in current technology is controllable large volume operation. To address this challenge, we have developed an experimental setup for flow through electroporation with online regulation of the flow rate with feedback control. We have designed a modular serial flow-through co-linear chamber with a smooth inner surface, the uniform cross-section geometry through the majority of the system’s length, and the mesh in contact with the electrodes, which provides uniform electric field distribution and fluid velocity equilibration. The cylindrical cross-section of the chamber prevents arching at the active treatment region. We used mathematical modeling for the evaluation of electric field distribution and the flow profile in the active region. The system was tested for the inactivation of *Escherichia coli*. We compared two flow-through chambers and used a static chamber as a reference. The experiments were performed under identical experimental condition (product and similar process parameters). The data were analyzed in terms of inactivation efficiency and specific energy consumption.

## Introduction

Straightforward and simple methods for cell membrane permeabilization are required in different fields of biotechnology, wastewater treatment, medicine, pharmacy, agronomy, and food processing. In a living cell membrane integrity plays a crucial role for cell homeostasis and the exchange of molecules with the surroundings. Nevertheless, in some biotechnological, medical, or agronomical applications the barrier of the cell membrane has to be overcome in order to administer drugs or genes to the target cells while preserving cell viability. In other applications, the extraction of specific compounds from living cells is desired. Sometimes, intentionally provoked cell death is the final goal, such as for tissue ablation in medicine or the elimination of undesired microorganisms from food and wastewater in biotechnology. Among different physical methods enabling cell membrane permeabilization, pulsed electric field treatment (PEF), which causes cell membrane electroporation, has gain attention for its reliability, simplicity and effectiveness^1–6^.

Electroporation is a phenomenon observed in living cells exposed to electric pulses of micro to millisecond durations with electric field amplitudes in the range from V/cm to several tens of kV/cm. The phenomenon can be described as a dramatic increase in membrane permeability caused by hydrophilic pore formation in the lipid bilayer of the cell membrane. The external electric pulses induce transmembrane potential, resulting in structural rearrangements of the membrane phospholipids^5–8^. Hydrophilic pores form only in a small fraction of the membrane exposed to the electric field. The induced potential depends on the cell shape and size, the strength of the applied electric field, and the angle between the direction of the electric field and the selected point on the cell surface. The induced transmembrane potential is higher on larger cells, and electroporation occurs at the poles of the cell facing the electrodes where the induced transmembrane potential is maximal^6, 9–11^. The electroporation can be either reversible or irreversible, depending on the parameters of the electric pulses. For electropermeabilization, a critical transmembrane potential between 200 mV and 1 V is required^6, 7, 10^. The increment of the potential can proceed until the second critical value is achieved leading to irreversible electroporation, membrane disintegration, and cell death^2, 5^.

Electroporation has been used for manipulation of different cell types and has been applied to bacteria, yeast, plant and animal cells^1, 3, 9, 12–15^.

For investigations of basic mechanisms and for laboratory scale operation, small volume electroporation in stationary chambers is sufficient and preferable, because all the cells are exposed to equal pulse parameters^16^. In such cases, the sample is typically restricted to volumes smaller than one ml; however, larger biotechnological and industrial applications require large volume processing^14, 17, 18^ and continuous flow electroporation. Most efforts for the optimization of large volume operation have been made in the field of food preservation for the inactivation of pathogen or spoilage microorganisms in liquid foods^11, 13, 18–24^. Some attempts have also been made for clinical wastewater treatments^25, 26^ and for gene delivery in mammalian cells or bacterial transformation^1, 17, 27, 28^.

The effectiveness of electroporation depends on the process parameters, product parameters, and cells’ intrinsic characteristics. The process parameters are electric pulse parameters, temperature of the treated sample and in continuous systems the flow rate of the sample. The product parameters are electroporation medium composition, conductivity, pH, osmolality, water availability, and the particle size in the sample. The intrinsic characteristics of the cell are growth conditions and physiological state, growth phase, stress tolerance, and recovery ability^1, 6, 20–22, 29–33^.

Pulsed electric field treatment is a safe method and US Food and Drug Administration (FDA) released a letter of no objection for PEF^32^ as an alternative method for food preservation^33^. From the industrial standpoint, the energy consumption required for PEF treatment is an important aspect to be taken into account. Some authors consider specific energy input as a reliable prediction factor for microbial inactivation^13, 18, 20^. However, further optimization can be made by incorporating the basic knowledge of electroporation accumulated in biomedical applications to PEF treatment^5^. Specifically, the electrical energy failed to predict the electroporation efficiency of animal cell^34^ and similar observations were also reported by some authors for the inactivation of bacteria^35^. Furthermore, large-volume operation relevant for industry requires the design of flow through treatment chamber and evaluation of flow parameters to obtain controllable and reliable electroporation of all the cells. Different types of flow electroporation chambers have been proposed^11, 18, 36, 37^.

In 2000 a review on pulsed electric field treatment published for US Food and Drug Administration (FDA) contained a comprehensive comparison and description of the treatment chamber designs^32, 33^. A treatment chamber is an important part of the pulsed electric field processing and comprises of two electrodes fixed by insulator that at the same time provides a container for the treated sample. Different electrode configuration are possible: parallel plates or wires, concentric cylinders or rod-plate electrodes. Parallel plates provide uniform electric field distribution, while concentric cylinders provide smooth and uniform fluid flow and are ideal for industrial applications^11, 32, 38^. Variety of static and continuous treatment chambers were developed by different authors as reviewed in detail in refs 11 and 33. The effective continuous flow chambers were developed by Dunn and Perlman in 1987, Matsumoto *et al*., 1997 and Zhang 1996 (described in refs 11, 33, 39). Most common chamber designs can be classified into three main types: planar/linear, coaxial and co-linear^18, 40, 41^. Additionally, treatment chambers can be classified based on the direction of the electric field application and the flow in cross-field; the directions are normal to each other, or co-field with parallel directions. A planar cross-field chamber design produces homogeneous electric fields and consists of two parallel electrodes and the treated sample flowing between them. The electric field in the active region is estimated as voltage to distance ratio (U/d). The drawback of this design is sealing of the chamber and arching at the interface of insulator, electrode and liquid sample. The co-filed chambers can be coaxial or co-linear. The coaxial chambers solve the problems of planar chambers and consist of two concentrically placed electrodes. Their drawback is non-homogeneous electric field distribution, the positioning of the electrodes, and restricted flux path. The co-linear treatment chambers comprise of two ring electrodes of the same diameter positioned next to each other and placed on the same axis. Among all types of the chambers this design results in the most non-homogeneous electric field distributions that can be corrected by geometry and insulator design^42–44^ or by placing metal meshes attached to the electrodes perpendicular to the fluid flow^45, 46^. The insulator geometry and metal mesh homogenize the applied electric field. However, a problem of high electric field intensity in the contact zone of the insulator, electrodes, and liquid sample persists and may cause arching and undesirable electrochemical reactions, consequently causing damage to the electrodes and the treated sample^43, 44^. The ongoing open question in continuous flow electroporation remains uniform electroporation for all cells in the flux, homogenous electric field distribution, and stable flow with uniform velocity distribution. Because in practice, this is difficult to achieve simultaneously some optimal compromise must be made. To date insufficient attention has been dedicated to the flow rate control, which is determinant for the number of pulses received by each cell. Thus far, the number of pulses has been estimated by multiplication of the electric pulse frequency and the residence time of the product in the treatment chamber. The residence time of the product has been calculated as a ratio of the treatment volume of the chamber and the volumetric flow rate of the treated product^47, 48^.

The persisting limitation of pulsed electric field treatment technology remains the construction and availability of high voltage electric pulse generators^33^. To solve this problem fast flow-through microbial inactivation was proposed recently by using constant radiofrequency electric fields that require lower electric fields and could greatly simplify the design of the generators^37^.

The aim of our study was to address some of the persisting open questions in the continuous flow PEF treatment; the uniform electroporation by homogenous electric field distribution and uniform sample flow within the active region of the PEF chamber. The novelty of our approach is the on-line regulation of the flow velocity with the feedback control interesting for large-scale applications and mathematical modeling of electric field distribution and the flow velocity profile. From the literature review, we identified the missing information, the direct comparison of different treatment chamber types operating under identical process and product parameters. We selected the planar cross-field (linear), co-linear co-field (MSFT) flow through electroporation chambers and a stationary chamber which served as a reference. The drawback and the limiting factor of our system remains electric pulse generator capacity.

## Methods

### Description of the experimental setup

In our experiments, electroporation was carried out in three different electroporation chambers: a static one with 1 mm or 2 mm distance between aluminium electrodes, and two different flow through chambers: planar/linear cross field with 2,5 mm between electrodes^47, 48^ and newly designed co-field co-linear modular serial flow through device, abbreviated as MSFT^49^ with 2 mm electrode distance. The electrodes in both flow through chambers were made from stainless steel. Rectangular electric pulses were generated by electric pulse generator HPV-VG (Igea, Capri, Italy)^50^ or a prototype pulse generator developed at the Faculty of Electrical Engineering, University of Ljubljana, described in ref. 48. Electric pulse amplitude was set to 30 kV/cm for 1 mm or 15 kV/cm for 2 and 2,5 mm electrode distance, which was a maximum electric field strength deliverable by electric pulse generators. Electric pulse parameters were first tested in the static chamber due to the higher range of electric pulse amplitudes available at 1 mm electrode distance^50^ as indicated in Table 1. In flow through system, the repetition frequency was 1 and 10 Hz and the pulse number was 8 or 20 pulses with the pulse duration of 100 µs. In the MSFT device, which poses two treatment regions, we performed additional experiments to compare its effectiveness when operating in one or both treatment regions. Pulses were delivered in one treatment region as 8 or 20 pulses or in both treatment regions as 4 pulses in the first region followed by 4 pulses in the second region (pulsing protocol 4 + 4) or alternatively 10 pulses in each region (10 + 10 pulses). In an additional experiment, 20 pulses per active region, 20 + 20 pulses was also tested.

**Table 1:** Preliminary experiments for determination of electric pulse parameters for flow through electroporation system.

electric pulse parameters	inactivation log_10_(N/N_0_)
**amplitude**	
8 × 100 µs 7,5 kV/cm	−0,46 ± 0,23 (N = 3)
8 × 100 µs 15 kV/cm	−1,02 ± 0,62 (N = 5)
8 × 100 µs 30 kV/cm	−1,94 ± 0,72 (N = 16)
**duration**	
8 × 50 µs 30 kV/cm	−1,03 ± 0,13 (N = 3)
8 × 250 µs 30 kV/cm	−1,63 ± 0,46 (N = 3)
**number**	
20 × 100 µs 30 kV/cm	−3,51 ± 0,92 (N = 6)
24 × 100 µs 30 kV/cm	−3,38 ± 0,63 (N = 3)
48 × 100 µs 30 kV/cm	−4,56 ± 0,10 (N = 3)
24 × 100 µs 15 kV/cm	−1,24 ± 0,19 (N = 3)
48 × 100 µs 15 kV/cm	−2,37 ± 0,45 (N = 3)

Experiments were performed in stationary electroporation chamber with aluminium electrodes and commercially available electric pulse generator HPV-VG (Igea, Capri, Italy). Data are mean ± standard deviation. Number of independent experiments is indicated as N^50^.

**Figure 1:**
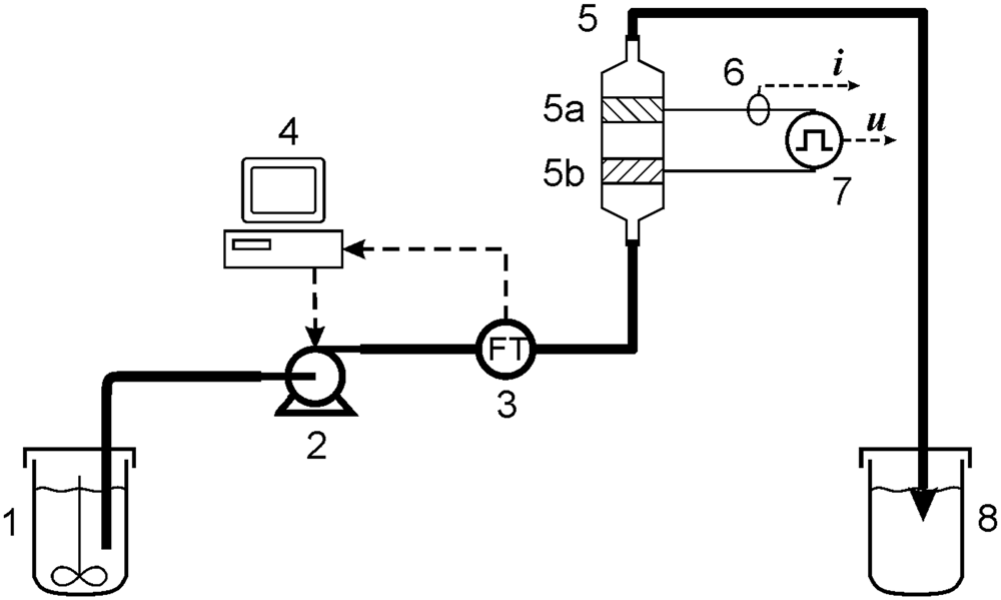
Experimental setup of continuous electroporation treatment. 1-sample, 2-pump, 3-flow sensor, 4-PC with flow control, 5*-MSFT with treatment regions a and b (exchangeable*), 6-current probe, 7-voltage probe, 8-treated sample of *E. coli* for plating on Petri dishes. *When linear chamber was used it replaced MSFT in position 5. The linear chamber is described in details in refs 47 and 48. Schematic drawing of chambers is given in Fig. 3.

The experimental setup for flow through electroporation is presented in Fig. 1. The suspension of *E*. *coli* (1) was pumped to the tubes by geared electric pump (Kavan 0190, Nuernberg, Germany) (2) and the flow velocity was monitored by flow-meter B.I.O-TECH (Vilshofen, Germany) (3). The flow rate was recorded and controlled by personal computer with the software for control, data acquisition and Matlab/Simulink (Mathworks, Inc., Natick, MA, USA) equipped with an NI USB – 6215 acquisition card (4) (National Instruments, Austin, Texas, USA). The electroporation chamber (5) was placed in vertical position, the pulses were recorded with an oscilloscope (Le Croy 9310 dual, New York, USA); current and voltage were monitored by current (6) (Le Croy AP015, New York, USA) and voltage (7) (Tektronix P6015A, Beaverton, USA) probes. The sample was collected in a sterile 1.5 ml Eppendorf tube (8) at three different points of the electroporation process. The flow rate was 0.44 ml/s for 8 pulses, and 0.18 ml/s for 20 pulses. The flow velocity profile was laminar (parabolic) in linear chamber while uniform in the active region of MSFT chamber. In the MSFT chamber operating in two active regions, the flow rate was 0.88 ml/s (4 + 4) and 0.35 ml/s (10 + 10) at the pulse repetition frequency 10 Hz. Estimated power corresponding to the peak during the pulses was 90 kW (24 A, 3,75 kV) for linear chamber and 60 kW (20 A, 3 kV) for MSFT device. The elimination of the air bubbles was obtained by vertical positioning of the treatment chambers, as described by others^47, 48^.

As a reference, we used a static chamber with defined electric pulse number received by each bacterium. The stationary chamber was a cuvette with two aluminium parallel plate electrodes (Eppendorf, Germany) with 2 mm distance between them.

### Features of modular serial flow through device MSFT

**Figure 2:**
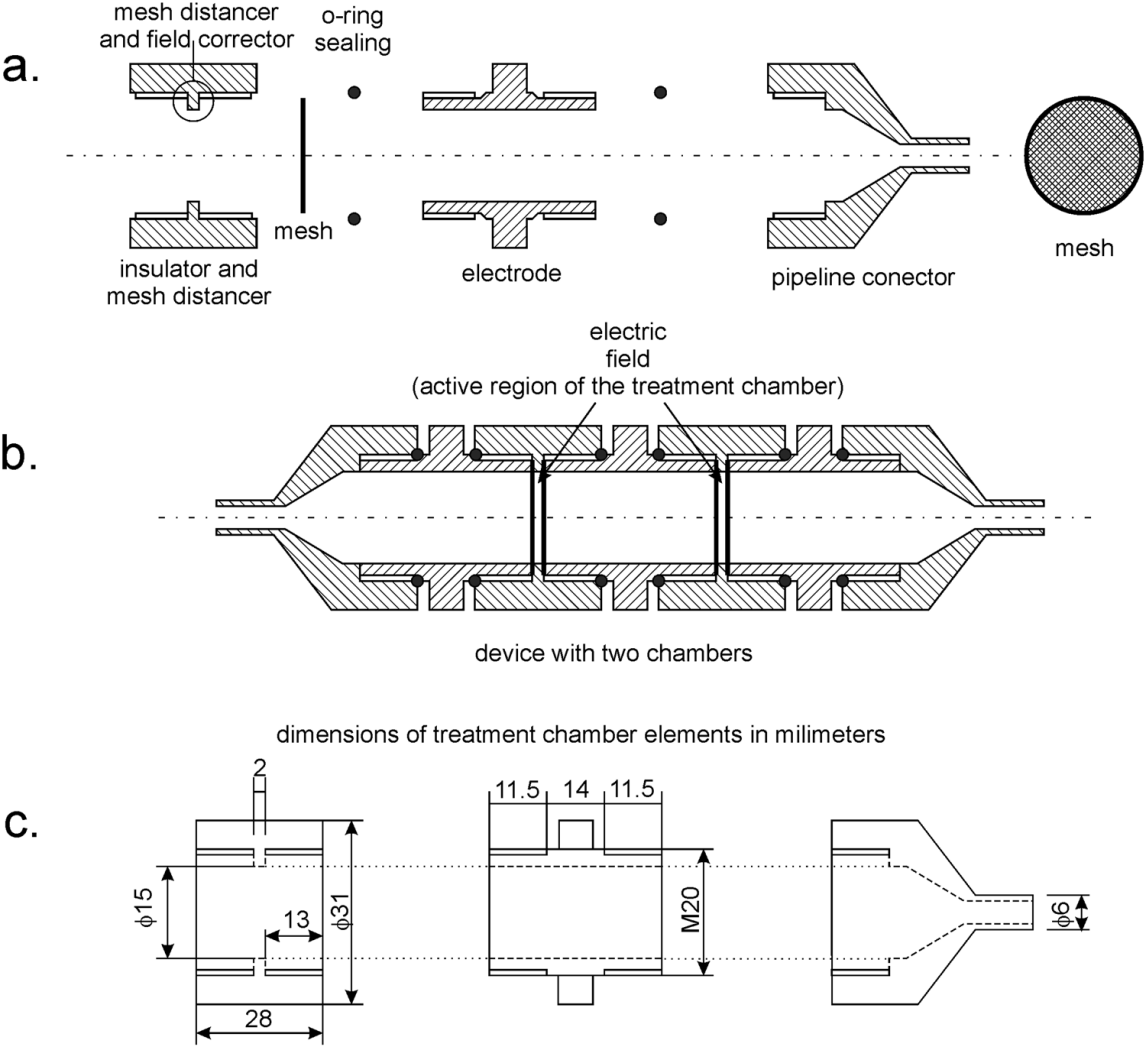
MSFT configuration with corresponding pipes, isolator shape, mesh electrodes and the position of the active treatment regions (**a**) specific insulator shape (**b**) longitudinal cross section of the chamber (**c**) dimensions of pipes and connectors.

The meshes are one of the key feature of the MSFT and have two important roles. They function as electrodes providing homogenous electric field distribution, and they equilibrate flow velocity distribution through the active regions of the MSFT. The mesh electrodes are removable, enabling replacement and adaptation of the mesh grids to the particle size of the treated sample (Fig. 2). The shape of the tube connectors at the flow entrance to the MSFT enables linear transition from the connecting tubes to the full chamber diameter (Fig. 2) and prevents the collection of the air bubbles within the device at vertical mounting. Reynolds numbers were calculated to estimate the flow patterns. They are ranging from 75 (using water flow properties at 20 °C) for the fastest and 30 for the slowest flow rates. This is well below the turbulent region, which usually starts above 1000^51^.

**Figure 3:**
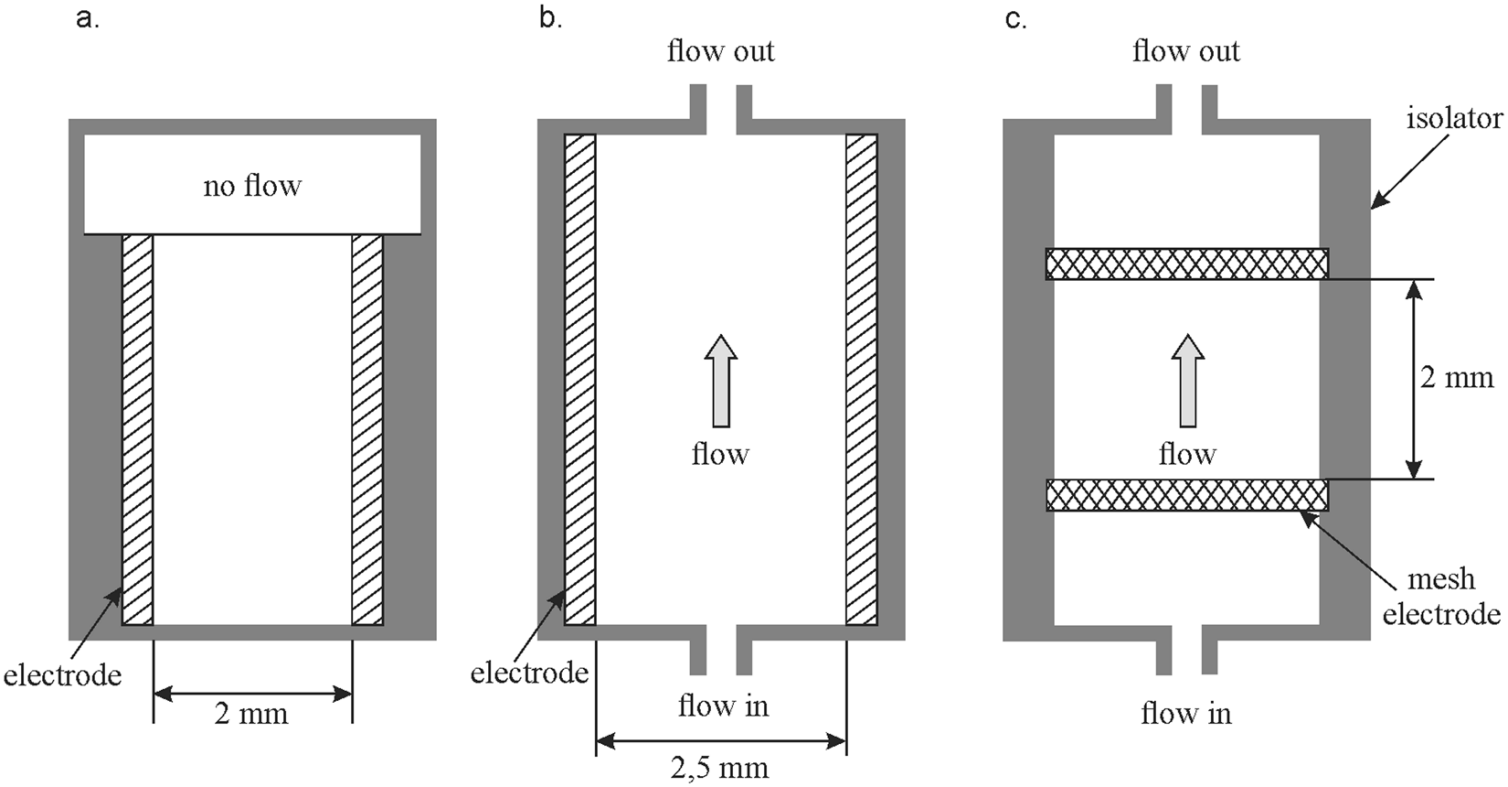
Schematic representation of three treatment chambers: (**a**) reference static chamber, (**b**) linear flow through chamber (**c**) modular serial flow through (MSFT) chamber with only one treatment region. The flow direction is indicated by arrow. The position of electrodes and the electrode distance defining the active treatment region are indicated in the drawing.

**Figure 4:**
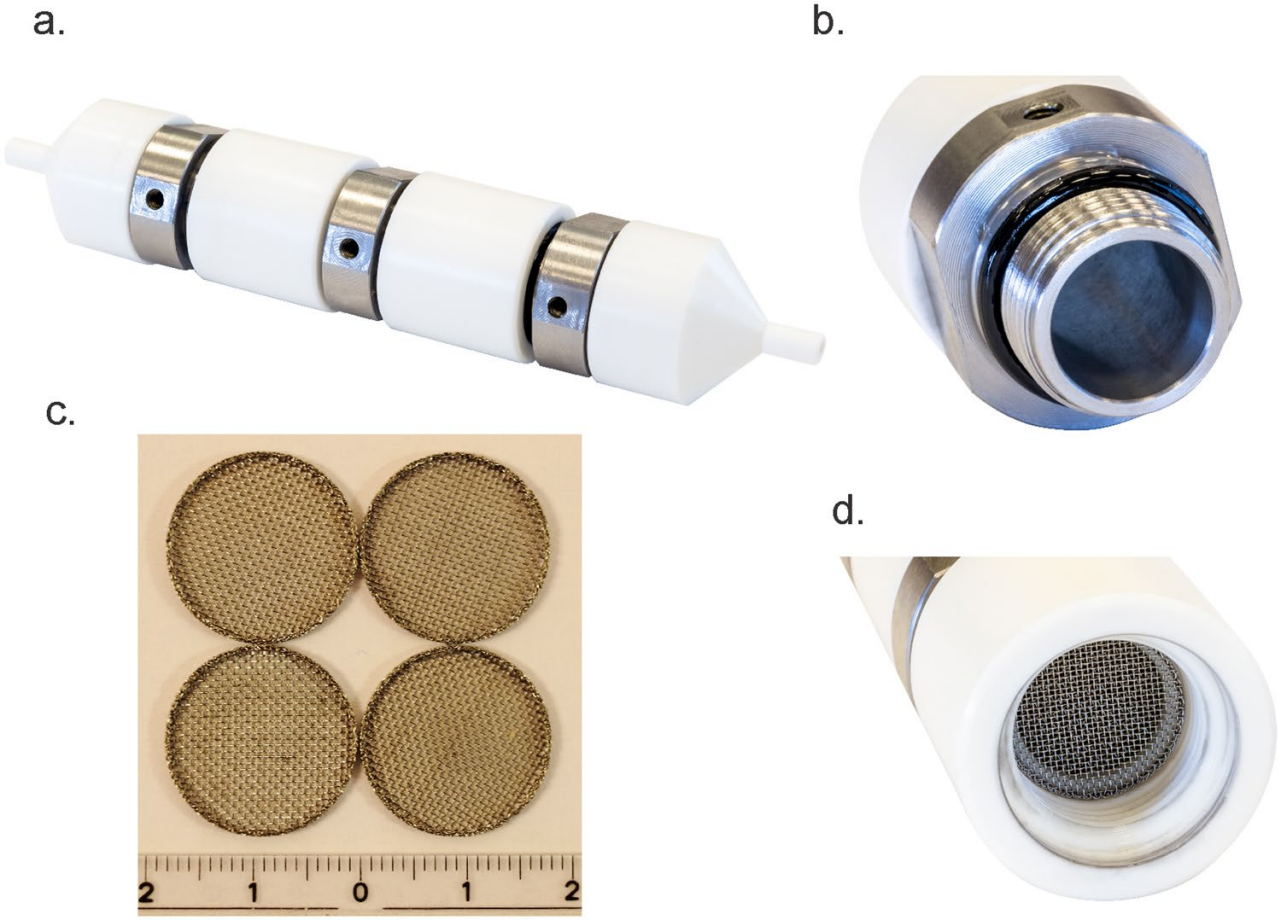
The structure of MSFT chamber (**a**) exterior of assembled chamber with pipelines connections, (**b**) the stainless steel meshes acting as electrodes in one of the two active treatment regions (**c**) the undamaged stainless steel mesh electrodes after PEF treatment with the rule with cm unit labels (**d**) smooth inner surface of the chamber.

**Figure 5:**
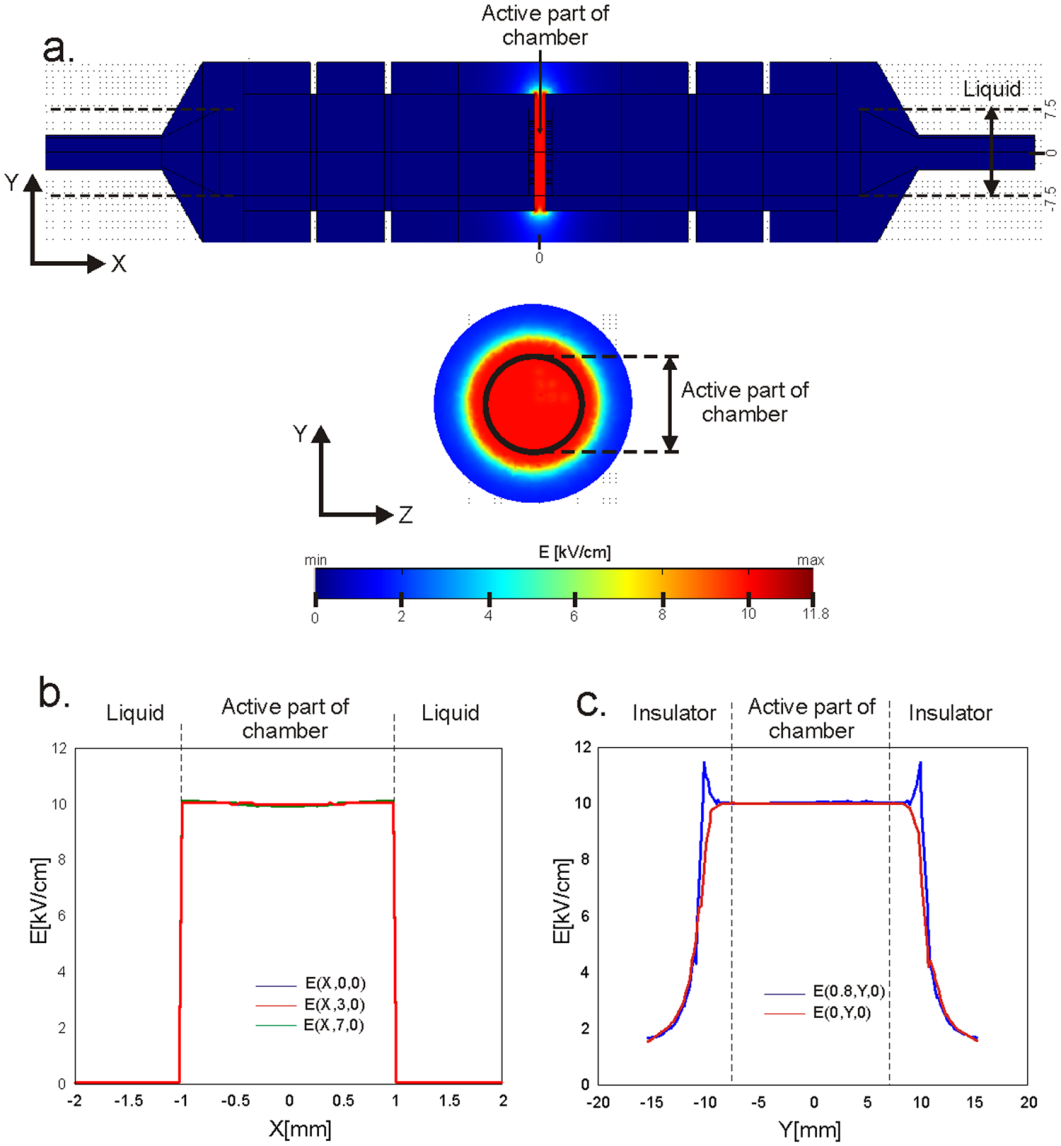
Calculated electric field distribution within the treatment chamber (**a**) in XY and YZ cross section plane, (**b**) along X axis and (**c**) along Y axis.

The active treatment region is located between the meshes (Figs 3 and 4), which are separated with the specifically shaped insulator (mesh distancer). The insulator shape in this region plays a key role in electric field homogenization. It eliminates the non-homogeneities and high gradients of the electric field strength at the edge of electrode-insulator interface of the planar capacitor formed with the two meshes (Fig. 5). This inhomogeneity and the high gradients of electric field strengths remain entirely within the insulator, providing a homogenous electric field in the active region of the sample treatment. The modular design of the device enables electroporation at higher fluxes at relatively low pulse repetition frequencies if chambers are added in series to the existing assembly.

We used the finite element analysis and the solver software package of Comsol Multiphysics (Comsol Inc., Burlington, USA) for the mathematical modeling of electric field distribution and fluid flow profile.

The resulting 3D geometry was built according to the dimensions given in Fig. 2. The flow direction (i.e. inlet and outlet) of the treated liquid sample is marked with the arrows in Fig. 3. Only one of the active regions between two conductive mesh electrodes is presented in the central part of the chamber. The model of the mesh electrodes was built as consistently as possible to the geometrical and material features of the mesh electrodes used (as indicated Fig. 4c). The mesh raster consisted of squares with 0.5 mm, while the width and height of the conductive part of the mesh electrode was 0.15 mm. The material properties and other parameters related to the modeled electrodes (stainless steel), isolator (Polytetrafluoroethylene - PTFE) and the liquid sample (water) were taken from the material library provided in the Comsol Multiphysics software.

The applied voltage was modelled as Dirichlet’s boundary condition assigned to the surface of the conductive mesh electrodes, while the body of the treatment chamber was mathematically separated from surrounding area by Neuman’s boundary condition. The medium of the treated sample was an isotropic electric conductor in a quasi-stationary electric current field with constant electric conductivity [S/m]. The calculated numerical results of electric field distribution within such a linear model, therefore, do not depend on the electric conductivity of the treated sample and are scalable by the value of the applied voltage. The theoretical considerations for modeling of electric properties and electric field distribution in electroporated treated samples are given in refs 52 and 53.

Mathematical modeling of the fluid flow profile was performed by using the Incompressible Navier-Stokes fluid dynamics application mode. The fluid flow profiles throughout the chamber configurations without and with the presence of mesh electrodes were modelled in order to evaluate the influence of the mesh electrodes on the flow profile within the active region. The fluid flow in the model was assumed to be laminar. A constant normal inflow velocity boundary condition was assigned to the inlet surface of the modeled chamber. The walls and the outlet of the model were set to have no slip boundary conditions and zero pressure, respectively.

### Experimental design and *E. coli* PEF treatment in three different chamber types

Three different electroporation chamber types were compared using one model organism: a non-pathogenic strain of *E*. *coli* K12 ER1821 (New England BioLabs, Germany). The *E*. *coli* culture was maintained on plates in LB Luria agar (Sigma-Aldrich, Germany). Before the experiment, an overnight liquid culture was prepared by inoculation of *E*. *coli* in Luria Broth medium (Sigma-Aldrich, Germany) in an Erlenmeyer flask. The culture was incubated at 37 °C for 16–18 h with continuous shaking in a water bath to obtain the culture in a stationary phase of growth (Kambič Slovenija). On the day of experiment, the concentration of bacterial suspension was determined using optical density measurement at 600 nm in a spectrophotometer (Eppendorf, Germany) and adjusted to a final concentration of 10^9^ CFU/ml. The bacterial culture was centrifuged at 4.2000 RCF for 30 min at 4 °C (Sigma 3–18, Germany) to obtain a cell pellet. The supernatant was removed and the pellet was resuspended in distilled water. The conductivity of bacterial suspension was determined with a conductometer (Metrel, Slovenia) and was 300.5 ± 16.5 µS/cm for an undiluted suspension. The temperature range of sample solution was between 20 and 30 °C, pH values were between 7 and 7.4. The suspension was diluted for electroporation experiments to a concentration of 10^7^ CFU/ml with conductivity of 4.7 ± 0.185 µS/cm. We performed parametric study of electric pulse parameters in stationary electroporation chamber with 1mm electrode distance. Next, with the obtained results, we selected electric pulse parameters for comparison of three different electroporation chambers. For those experiments the sample was divided into three parts: one for a stationary chamber that served as a reference and two for continuous flow chambers. The used treatment chambers are presented schematically in Fig. 3. Both a cross-field linear chamber and newly designed co-field MSFT were connected to flow control and feedback regulation as indicated in Fig. 1. The comparison of three chambers was performed in aseptic conditions as four independent experiments. The flow-through system was sterilized with 70% ethanol pumped through the system for ten minutes. Then the ethanol was removed from the system and the setup was washed with sterilized distilled water. Before electroporation, a sample of *E*. *coli* was run through the system to remove the residual water and descanted. A fresh sample was loaded in the system, exposed to electric pulse treatment, and electroporated bacterial suspension was collected in a separate sterile recipient (Fig. 1) for each pulse parameter and electroporation chamber. The conductivity and pH of the sample was measured after the treatment, and no changes were detected. The effectiveness of the PEF inactivation was determined by viable cell count 24 h after the experiment. The volume of the treated sample was 400 µl for the reference stationary treatment chamber. The sample from the flow-through setup was 1 ml collected in Eppendorf tube at three different time points of the electroporation process. The 100 µl of electroporated sample for a given treatment was serially diluted, and at least three dilutions of each sample were spread on plates with Luria agar (Sigma-Aldrich, Germany). At least two negative controls of untreated bacteria were prepared for each experiment. Plates were incubated for 24 h at 37 °C in an incubator (Kambič, Slovenia). Bacterial colonies were counted manually, and the results were expressed as colony-forming units per ml of the sample CFU/ml. The inactivation results were presented as log_10_ survival fraction S = N/N_0_ where N is the number of colonies of the treated samples, and N_0_ is the number of colonies in the untreated control. The inactivation was also presented regarding the energy consumption given as the inactivation per energy density given as energy per unit volume log_10_N/N_0_/J/ml.

The maximal temperature rise during electroporation was measured at the stationary chamber with an infrared thermometer (Fluke 62 Mini Infrared Thermometer, Fluke, USA). It was below 9 °C for undiluted cell suspension of *E. coli* at 10^9^ CFU/ml with 48 pulses of 100 µs and 30 kV/cm reaching a temperature of 37 °C at the end of the treatment. This data indicate that in our experimental conditions Joule heating was below this value, not affecting the survival of *E. coli*. The possible toxic effects of electrochemical compounds formed during electroporation were tested indirectly by exposing distilled water to 30 kV/cm. We exposed untreated bacteria to this water and we did not detect any toxic effects. In inactivation experiments no recovery was observed after electric pulse treatment due to the use of distilled water as an electroporation medium. Specifically, all bacteria were killed after the cell membrane was permeabilized due to the osmotic imbalance causing water entry into the living bacteria. The electrodes were not damaged after treatment and stayed intact even three years after they were used (Fig. 4).

The results obtained with static chamber and 1mm electrode distance are presented in a table as a means of different number of independent experiments ± standard deviation. The number of independent experiments is given in the table. For comparison of different chamber types the data are plotted as the actual values obtained at each of four independent experiments (n = 4). Independent experiments were performed in different dates from freshly prepared bacterial cultures.

## Results

### Mathematical modeling of electric field and flow velocity distribution in MSFT

The calculated electric field distribution in the XY and YZ central cross-sections of the chamber is displayed in the Fig. 5. Homogeneous electric field distribution (E = 10 kV/cm) is obtained within the active part of the chamber between the two mesh electrodes, while the electric field outside the active region (where there is no potential difference) is zero (Fig. 5b). The electric field within the insulation volume is highly non-homogeneous with the maximum value of electric field intensity E = 11.8 kV/cm panel C (Fig. 5).

**Figure 6:**
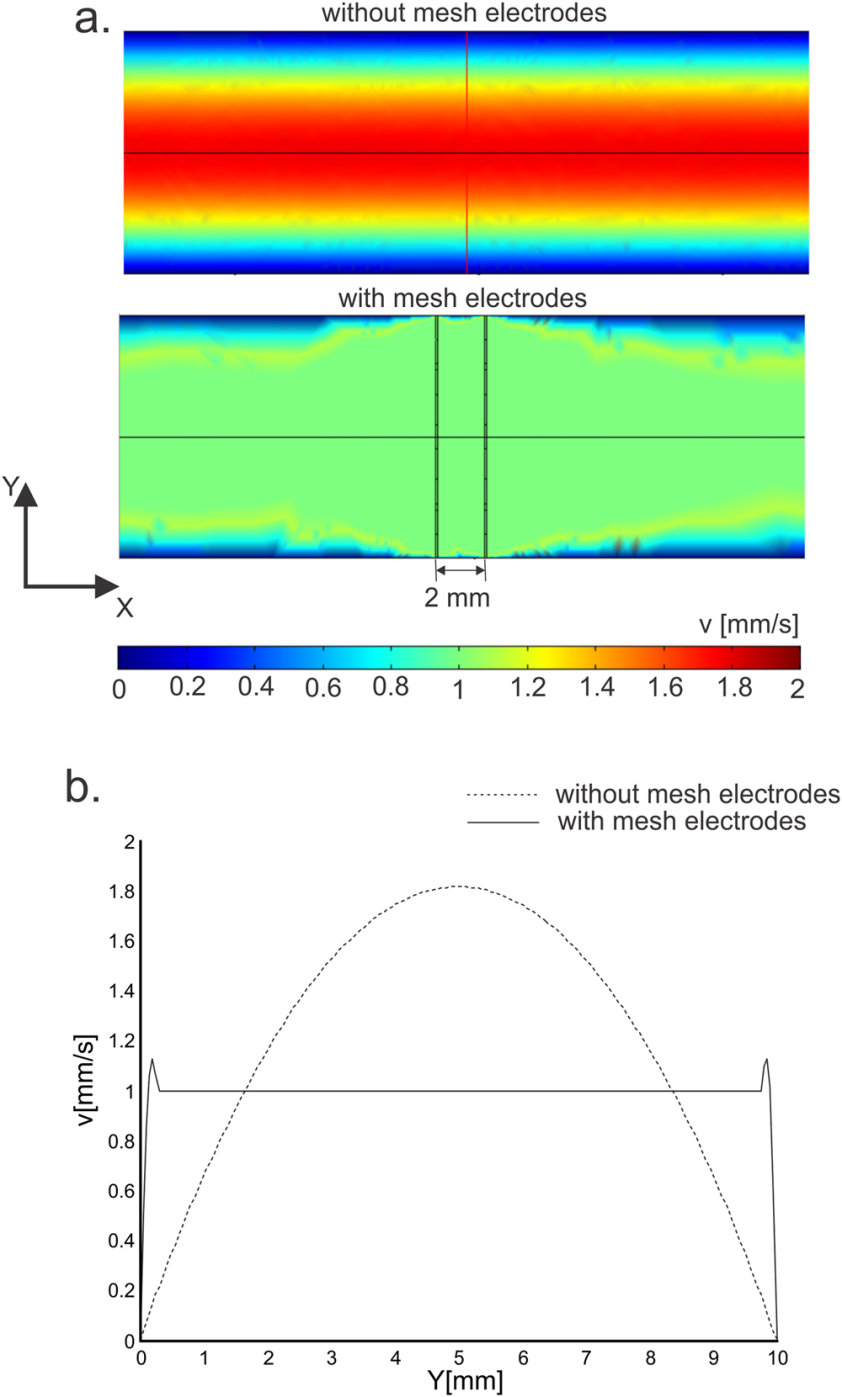
Calculated fluid flow velocity profiles within the treatment chamber without mesh electrodes and with mesh electrodes (**a**) v(X,Y) displayed in XY central cross section of the 3D model and (**b**) v(Y) along Y axis.

The results of fluid flow velocity profiles calculated throughout the modeled treatment chamber with and without the mesh electrodes are shown in Fig. 6. The calculated flow velocity profiles v(X,Y) in XY central cross section are displayed in panel A. The central v(Y) profile along the XY planes of both models are displayed in panel B. The initial velocity was v = 1mm/s. As expected, the fluid flow velocity profile throughout the treatment chamber without the mesh electrodes is laminar, with the minimum velocity in the near proximity of the wall and the maximum velocity in the center of the chamber (i.e. parabolic v(Y) profile). The insertion of conductive mesh electrodes resulted in a uniform fluid flow velocity profile within the active region between the mesh electrodes, as shown in Fig. 6.

### Experimental results of *E. coli* Inactivation in three electroporation chambers

A systematic study of electric pulse parameters on *E. coli* inactivation was performed in stationary chamber with electrode distance 1 mm. The aim of the experiments was to tested different electric pulse number, amplitude, and duration in the range available by given electric pulse generators. We tested 8 pulses of 100 µs duration and obtained −0,46 log_10_ inactivation for amplitude 7,5 kV/cm and −1,95 log_10_ inactivation for amplitude 30 kV/cm (Table 1). Based on the obtained data we tested the effect of pulse duration and pulse number on the inactivation. For pulse duration, we used only 30 kV/cm while for pulse number we used 15 and 30 kV/cm and 100 µs duration. The average inactivation (−1,45 at 15 kV/cm) and (−1,95 log_10_ at 30 kV/cm) was improved when we increased the pulse number. The best results, −4,56 log_10_ were obtained with 48 pulses at 30 kV/cm (Table 1).

In flow through experiments the distance between electrodes was 2 mm and 2,5 mm limiting the maximum pulse amplitude to 15 kV/cm. Higher electric pulse amplitudes are not available by our electric pulse generators.

**Figure 7:**
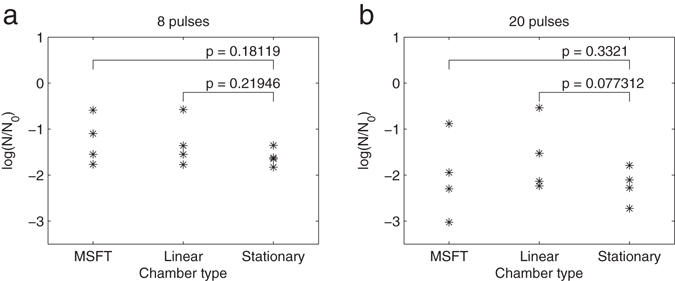
Inactivation of bacteria E. coli in three different electroporation chambers. Stacionary and two flow-through chambers: new colinear (MSFT) and linear with parallel plate electrodes. Number of pulses was (**a**) 8 pulses or (**b**) 20 pulses. Inactivation of *E. coli* is expressed as a log_10_ inactivation. Bacterial cells were exposed to rectangular electric pulses with the duration 100 µs, repetition frequency 10 Hz and electric pulse amplitude 15 kV/cm. Density of the treated sample was 10^7^ CFU/ml, number of independent experiments n = 4.

The experimental results of comparison of three electroporation chambers are shown in Fig. 7. On the pennel A, the inactivation for eight pulses 100 µs pulses with amplitude 15 kV/cm given as a log_10_ measure is shown. In the stationary chamber, we observed better inactivation in comparison with the results obtained by flow-through co-linear MSFT and linear chambers. We obtained −1.6 and −1.3 log_10_ reductions in stationary and flow chambers, respectively. On the pannel B, the results for twenty pulses are given: the average inactivation increased to −2.2 in stationary chamber and −1.6 and −2.0 log_10_ units in linear and MSFT flow chambers. At 20 pulses, inactivation in the linear chamber is worse than in the reference stationary chamber.

The MSFT chamber can operate in two modes, with one or two active treatment regions. The slightly higher inactivation rates (−1.3 ± 0.5 log_10_) were observed when cells were exposed to eight pulses in one treatment region compared to 4 + 4 pulses in two active regions (−1.1 ± 0.5 log_10_). A similar pattern was observed for twenty and 10 + 10 pulses (−2 ± 0.8 log_10_ vs. −1.4 ± 0.6 log_10_) respectively. A −2.74 log_10_ inactivation was recoded in the experiment applying 20 pulses in two active regions (20 + 20).

**Figure 8:**
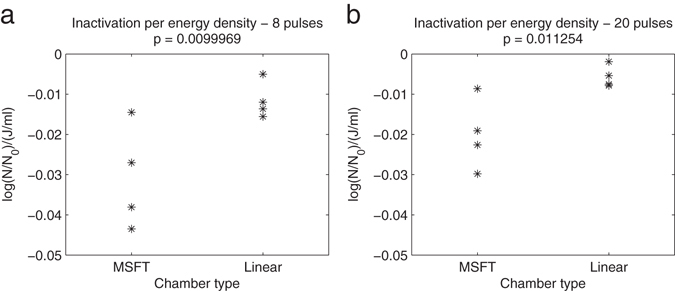
Energy consumption for inactivation of *E. coli* in two flow through chambers. Inactivation is expressed as a log_10_ inactivation per energy density given by energy per unit volume. (**a**) Bacterial cells were exposed to 8 or (**b**) 20 rectangular electric pulses. The duration was 100 µs, repetition frequency 10 Hz and electric pulse amplitude 15 kV/cm in MSFT and linear flow through chambers. Cell concentration of the treated sample was 10^7^ CFU/ml number of independent experiments n = 4.

The inactivation was evaluated also regarding the energy consumption, which was given as the inactivation per energy density given as energy per unit volume [log(N/N_0_/(J/ml)]. The results for the two flow-through chambers are given in Fig. 8. This comparison shows better results obtained with MSFT chamber than with the co-linear chamber.

## Discussion

The disadvantage of the continuous flow chambers, especially in the co-linear type, is the electric field inhomogeneity and uneven electroporation conditions for individual cell in the flow. To deal with this issue, we designed a new electroporation chamber, addressed the problem of flow velocity control and evaluated different chamber designs for defined product and process parameters.

A new co-linear modular serial flow through device (abbreviated as MSFT) is a co-field chamber enabling controllable, uniform flow electroporation for laboratory scale treatment. In general co-linear flow through chamber design results in the most non-homogeneous electric field distributions among all chamber types^42–46^. The main advantage of MSFT design over other co-linear chambers was the position of the mesh electrodes and shape of the insulator. The insulator is fixing the electrodes and at the same time defining cylindrical active treatment region with smooth inner surface (Figs 2 and 4). The meshes inserted in the specifically shaped insulator are perpendicular to the flow (Fig. 4) and have two functions: provide uniform flow and homogeneous electric field distribution within the active treatment region.

For uniform electroporation in the flow, each cell should receive the defined number of electric pulses. The number of pulses affect inactivation level at given electric field strength (Table 1)^50^. In continuous flow chambers, the number of pulses depends on the residence time of the sample in the treatment chamber and is determined by the volumetric flow rate^47, 48^. To improve controllability of delivered pulse number, we integrated on-line monitoring of the flow rate and the feedback regulation in our continuous treatment system. Such control compensated for variations of average flow velocity in experimental setup (Fig. 1) for both flow through treatment chambers. With uniform velocity of the flow in the entire system, the number of pulses received by each cell depends further on the flow velocity profile in the active treatment region. In linear chamber (Fig. 3) flow profile was laminar i.e. had two times higher velocity in the center than at the flanks. On the contrary, uniform flow velocity distribution within the active treatment region (Fig. 6) was obtained in MSFT chamber by insertion of the mesh perpendicular to the flow (Fig. 3). In theory, the equalized flow velocity profile within the active treatment region minimizes the variance in number of pulses received by the cells. In practice, this means smaller differences between stationary and continuous flow chambers. We could not confirm this assumption in our setup due to the limitation of electric pulse amplitude to 15 kV/cm, but the tendency was observed with higher pulse numbers (Fig. 7). At twenty pulses the average inactivation rate was −2,22 log_10_ in stationary, −2,02 log_10_ in MSFT and −1,61 log_10_ in linear chamber.

On-line control of the flow velocity was the advantage over simple estimation of number of pulses delivered per treated cell described in the literature^47, 48^. In our opinion, the lack of reliable flow regulation could explain why the batch laboratory setup comprising of a stationary chamber has been considered to be a better choice for uniform electroporation thus far. Specifically, stationary chambers ensure uniform treatment conditions for all the cells in the treated volume. The non-uniform flow is a special issue in a large transition zones with undefined electric field strength. Therefore, co-linear chambers were, in principle, less effective for microbial inactivation in comparison with cross field and coaxial chambers^42^. In our setup, co-linear MSFT chamber was at least as effective as cross-field linear and reference stationary chamber. Similar performance of all treatment chambers (Fig. 7) indicate that on-line control of the flow velocity could benefit the translation of the results from stationary chamber to pilot scale flow systems. The uniform velocity profile of the flow, known average velocity, and homogeneous electric field distribution are three prerequisites for the effective and predictive regulation of electroporation relevant for industrial scale PEF treatment. MSFT chamber has smooth inner surface (Fig. 2) does not contain large transition zones (Fig. 4) with undefined flow (Fig. 6) and electric field (Fig. 5) regions. The flow velocity in MSFT chamber depended on the number of applied pulses and was between 0,44 ml/s for eight pulses and 0,18 ml/s for 20 pulses applied at 10 Hz. This values were in the range described by Alkhafaji *et al*.^45^ for of 200 Hz, and reported by Geng *et al*.^28^. Some flow characteristics in the system depend on the pump, therefore to prevent pulsing flow, especially at a lower number of pump rotations we did not use peristaltic pump for sample circulation.

Undefined electric field regions in the MSFT chamber were prevented by specific insulator shape in function of electric field corrector (Figs 2 and 5). The homogenization of the electric field in co-linear chambers has been proposed previously by different solutions including conductive mesh electrodes and different insulator shapes in a diverse flow chamber design^42–46^. In those solutions the flow rate was modified by protrusions in the inner surface of the chamber and uniform electric field distribution over the entire active region has not been provided^11, 42–46^. In spite of current limitations due to insufficient electric pulse amplitudes available by the generators, the advantage of MSFT treatment chamber was smaller energy consumption required for given inactivation of *E. coli* compared with linear chamber (Fig. 8).

Another contribution of our study was experimental evaluation of distinct types of electroporation chambers under defined process and product parameters with known cells’ intrinsic characteristics. During the inactivation of *E. coli* pH of cell suspension stayed in range from 7 to 7.4 and temperatures from 20 to 30 °C were. Those values are within environmental conditions suitable for the growth of for this rod-shaped Gram negative bacteria living in the temperatures from 7 to 46 °C and the pH from 4 to 9^22^ and were not affecting inactivation. In our experiments, two-log_10_ reduction of *E. coli* was obtained with relatively low electric pulse amplitude (15 kV/cm) was due to limitations of electric pulse generators. Unavailability of high voltage electric pulse generators for PEF treatment^37^ is preventing systematic studies and has been the limitation for commercial application since the publication of the FDA report in 2000^32^. Differences in process parameters reported in literature^33, 37, 39^ are caused by the specific characteristics of electroporator prototypes and treatment chamber designs available at given institution. The main goal in the PEF treatment has been efficient microbial inactivation and according to our knowledge, no attempts have been made to use identical process and product parameters to compare different chamber designs. To make a step forward to a systematic study we defined process parameters available by our electric pulse generators (Fig. 1 and Table 1) and treatment chambers (Fig. 3) while keeping constant product parameters. Constant product parameters were maintained by defined cell density of *E. coli* and distilled water as electroporation medium avoiding the uncontrollable effects of cell number or medium components on inactivation. In distilled water, all electroporated bacteria died due to osmotic imbalances provoked by water uptake through permeabilized cell membrane. For evaluation of our results, we selected papers that used the same model organism and comparable range of electric pulse amplitudes; however, the main differences were in process parameters such as: electric pulse number, duration, repetition frequency, flow rates, electrode configurations and experimental setups. The product parameters such as medium composition (i.e. liquid food composition), conductivity, pH, osmolality, temperature, water availability and the particle size in the sample that affect electroporation (inactivation)^33, 38, 54^ were also diverse. Even though the two-log reduction obtained in our study is not sufficient for any practical application in food industry, the comparison with the data reported in literature^1, 6, 20–22, 29–31^ indicate that our results are at least in the same range. *E. coli* treated in co-field treatment chambers connected in series was reduced for 0.5 log_10_ units. This inactivation was obtained with significantly shorter pulse duration, higher repetition frequency, and electroporation medium consisting of beef extract, glucose, and peptone^13^. Ravishankar *et al*.^55^ obtained 1 log_10_ reduction applying similar number of pulses, to a gel with low pH in a stationary treatment chamber. The electroporation medium composition is crucial for recovery of the treated cells. In our opinion, it is likely that antioxidants present in electroporation media (food products, beef extracts, etc.) protect bacteria and enable recovery after treatment similarly to observations reported in mammalian cells^56^. Higher inactivation of undesired microorganisms in food with PEF treatment were obtained with higher electric pulse amplitudes (30 kV/cm or more)^19, 33^ and when PEF treatment was combined with other non-thermal methods for food preservation^24^.

To conclude, we made the comparison of different treatment chambers operating at the same experimental conditions (equal product parameters and similar process parameters) in our experimental set up. The online regulation of the average flow velocity provided a well-controlled environment within the active treatment regions of both flow through chambers, comparable to that of a stationary chamber. The main advantage of the MSFT device was uniform electric field distribution combined with uniform distribution of flow velocity in the active treatment region. The key elements in MSFT chamber were mesh electrodes and the insulator with specific shape. This insulator functioned as electric field corrector in the active treatment region and as a mesh distancer fixing the electrodes in appropriate position. The meshes inserted perpendicular to the flow direction provided uniform flow velocity profile and homogenous electric field. The MSFT chamber required lower total specific energy consumption and consequently caused lower heating of the sample compared with cross-field linear chamber. MSFT chamber is modular and can be enlarged by several active areas set up in series merely by adding insulator-electrode combinations. The main drawback in our study was the limiting capacity of electric pulse generators. For further experiments on inactivation relevant to food industry pulse amplitude of 15 kV/cm is too low, at least 30 kV/cm is required.
